# Peccavi

**Published:** 1862-03

**Authors:** 


PECCAVI.

We don't know the origin of this word, and perhaps not its exact meaning. Tradition intimates that a certain ancient heathen race, somewhat inclined to military achievements, used it instead of "throwing up the sponge," or "hallooing *enough*." Do you ask, reader, why we use it? "O. U. C." we (I) *have sinned.* Or, if you don't see it now, turn to our Philadelphia correspondence in the present number, and you will see it. "O. U. C." it now, do you? Well, then, help us to find out exactly in what the sin consists; for repentance is one of the "exact sciences," and needs a definite foundation.

Our correspondent charges us with acting toward the Faculty of the Pennsylvania College in a manner "unwarranted," "unmerited," *unjust* and *insulting*, through "ill feeling" at that, besides *disjointing* and *perverting* his language; and he notifies the jury that is to try us (the professional public) that, if they return a verdict of "Not guilty," they are to be branded as *prejudiced*.

We feel badly about this. Why shouldn't we? for each member of said Faculty is not only a professional, but a *personal friend.* And we would feel worse, if the charge had any foundation outside of the, for the time, "disjointed and perverted" imagination of our correspondent. But, "O. U. C." the charge is "as unwarranted as unmerited;" and nobody else is likely to discover symptoms of disrespect toward *anybody*, unless it be disrespectful for an editor to differ in opinion with a correspondent.

But let us analyze and compare:—Our correspondent alluded to the Faculty of the Pennsylvania College. We did, too. Was that "unwarranted?" He spoke rather favorably of the Faculty.

We did, too. Was that "*unmerited?*" He intimated that the Faculty was able. We couldn't help but coincide. Was that a display of "ill feeling?" We went farther, and intimated that, with all their talents, they were not superior to Professors Taylor, Chapman, Taft, Richardson and Atkinson. Does that amount to positive injustice? We intimated that Professors Austen, Bond, Wright, Piggot, Johnston and Gorgas were at least their equals in "ability." Is that "positive insult?" Well, scarecely! is the spontaneous verdict of "every unprejudiced mind."

"But Brutus says he was ambitious; and Brutus is an honorable man."

Now, reader, don't be alarmed. Nobody is going to regard us as less friendly to the brethren of the Pennsylvania College than is our correspondent. And we have no fears that our friendship will lack that desirable ingredient, mutuality. W.

